# Adaptive sensory coding of gaze direction in schizophrenia

**DOI:** 10.1098/rsos.180886

**Published:** 2018-12-12

**Authors:** Colin J. Palmer, Nathan Caruana, Colin W. G. Clifford, Kiley J. Seymour

**Affiliations:** 1 School of Psychology, UNSW Sydney, Sydney, New South Wales 2052, Australia; 2 Department of Cognitive Science, Macquarie University, Sydney, New South Wales 2109, Australia; 3 ARC Centre of Excellence for Cognition and Its Disorders, Sydney, Australia; 4 School of Social Sciences and Psychology, Western Sydney University, Sydney, New South Wales 2150, Australia

**Keywords:** schizophrenia, gaze perception, adaptation, aftereffects, normalization, visual processing

## Abstract

Schizophrenia has been associated with differences in how the visual system processes sensory input. A fundamental mechanism that regulates sensory processing in the brain is gain control, whereby the responses of sensory neurons to a given stimulus are modulated in accordance with the spatial and temporal context. Some studies indicate an impairment of certain cortical gain control mechanisms in schizophrenia in low-level vision, reflected, for instance, in how the visual appearance of a stimulus is affected by the presence of other stimuli around it. In the present study, we investigated higher-level, social vision in schizophrenia, namely the perception of other people's direction of gaze (i.e. a type of face processing). Recent computational modelling work indicates that perceptual aftereffects—changes in perception that occur following repeated exposure to faces that display a specific direction of gaze—are indicative of two distinct forms of gain control involved in the coding of gaze direction across sensory neurons. We find that individuals with schizophrenia display strong perceptual aftereffects following repeated exposure to faces with averted gaze, and a modelling analysis indicates similarly robust gain control in the form of (i) short-term adjustment of channel sensitivities in response to the recent sensory history and (ii) divisive normalization of the encoded gaze direction. Together, this speaks to the typical coding of other people's direction of gaze in the visual system in schizophrenia, including flexible gain control, despite the social–cognitive impairments that can occur in this condition.

## 1. Introduction

People with schizophrenia exhibit a range of perceptual and neural responses to visual stimulation that differ from individuals without this diagnosis. Identifying systematic differences in how sensory information is processed in the brain has been a key target of schizophrenia research in the past decade (e.g. [1–5]). This research has been performed partly to shed light on perceptual disturbances with direct clinical importance, such as the tendency to experience visual or auditory hallucinations, and partly as a handle on physiological or information processing differences that may have impact across sensory and higher-level functions. A fundamental sensory mechanism that has been explored in this regard is *gain control*, whereby the neural processing of a given stimulus is modulated in accordance with the sensory context. For example, the responses of a visual neuron to a stimulus presented within its receptive field can depend on whether it has recently been exposed to the same type of stimulus. In general, the way in which sensory input from a particular part of the visual field is processed depends on both the *spatial context*, such as visual input from adjacent regions of the visual field, and the *temporal context*, such as the very recent history of sensory stimulation. Both spatially and temporally sensitive gain control mechanisms are prevalent across different regions of the visual hierarchy and in other sensory modalities, enacting a set of computational functions that promote robust and efficient sensory coding (e.g. [6,7]). Gain control can be instantiated by shorter-range connections within cortical areas (e.g. inhibitory GABAergic neurotransmission [8]) as well as longer-range neuromodulation (e.g. excitatory top-down projections [9]). Probing the function of gain control mechanisms in schizophrenia is therefore important to understanding perceptual and neural data across different perceptual contexts, and for linking the reported perceptual differences (including hallucinations) to underlying neural and computational mechanisms [1].

Research on the function of gain control mechanisms in schizophrenia has commonly focused on how visual perception is modulated by the spatial context. Key evidence for a form of *impaired gain control* in schizophrenia is reduced susceptibility to the ‘contrast–contrast’ illusion [10]. In this illusion, the contrast of a textured patch appears lower when it is surrounded by a high-contrast texture compared to when it is presented against a blank background. The perceptual effects of the surrounding area on the target patch are tightly linked to a corresponding suppression of neural responses to the target in V1, as recorded with functional magnetic resonance imaging (fMRI) [11]. Individuals with schizophrenia tested by Dakin *et al*. [10] showed a clear tendency towards a more veridical perception of the test patch compared to both healthy controls and controls with other psychiatric conditions. In other words, perceived contrast for a particular part of the visual field was relatively unaffected by the surrounding visual context. This finding has since been replicated in several other samples [12–14], though Barch *et al*. [15] report partially conflicting data. In healthy controls, similar effects of the immediate spatial context on the perception of a visual target occur for luminance, orientation, size and motion. However, the evidence that contextual modulation is reduced in schizophrenia for these other perceptual properties is less consistent. For instance, Seymour *et al*. [16] found that orientation-dependent surround suppression of neural responses in V1, as measured with fMRI, is reduced in patients with schizophrenia (see also [17]), but, behaviourally, two studies have found that contextual modulation of perceived orientation is robust in this condition [13,14]. Robust contextual modulation of perceived luminance and motion has also been reported in schizophrenia, and there are mixed findings for perceived size [13,14,18]. These results highlight the value of examining canonical sensory mechanisms like gain control across perceptual domains, and correspondingly, distributed brain systems, to define the scope of visual processing differences in schizophrenia.

In the past decade, there has been increasing research on the visual processing of *higher-level* and *social* aspects of our perceptual experience, such as our visual perception of faces and biological motion. In schizophrenia, these social aspects of visual perception are important to study due to the emerging evidence that social difficulties contribute significantly to functional outcomes in this condition [19–22] and the social content of delusions and hallucinations. Canonical sensory mechanisms like gain control, the effects of which are established in lower-level vision, are also relevant to higher-level visual processing, but are largely unexplored in the latter context in schizophrenia. The present paper focuses on *perceived gaze direction*; that is, how our sense of where another person is looking is extracted from the sensory features of their face. There is evidence from both primate electrophysiology [23,24] and human fMRI [25,26] that higher-level visual pathways in the temporal cortex, particularly the anterior region of the superior temporal sulcus (STS), contain cell populations that respond selectively to the gaze direction of a seen face. Importantly, there is psychophysical evidence that the representation of perceived gaze direction across gaze-selective sensory neurons involves the operation of two distinct gain control mechanisms: *sensory adaptation*, whereby neural responses are dependent upon the recent history of stimulation, and *divisive normalization*, whereby neural responses are dependent upon the current activity of functionally related cell populations.

*Sensory adaptation* to gaze direction occurs when prolonged exposure to a particular direction of gaze produces a temporary shift in perceived gaze direction away from the direction adapted on [27,28]. For example, viewing a series of faces with a high prevalence of far *leftwards* gaze directions can make faces with other directions of gaze appear to be looking more *rightwards* than they really are. In general, perceptual aftereffects are commonly understood in terms of the *population coding* of stimulus properties [29], where a property of the world (e.g. edge orientation) is represented in terms of the relative activity of cell populations that are tuned differently along this dimension (e.g. cells tuned to more cardinal or more oblique orientations). Selective changes in the responsiveness of sensory channels following exposure to stimulation (e.g. habituation of a specific neuron tuned to vertical orientations following prolonged exposure to its preferred stimulus) can account for changes in relative activity across the channel population in response to a given stimulus, and thereby temporally dependent changes in perception. We recently found that the psychophysical effects of adaptation to horizontally averted gaze can be accounted for by a model of the gaze system consisting of three sensory channels, tuned broadly to leftwards, direct and rightwards gaze, respectively, where the gain on each channel is selectively reduced following adaptation in proportion to that channel's sensitivity to the adapting stimulus [30–33]. Importantly, these effects still occur when adaptation and test faces differ significantly in their lower-level features, suggesting that the perceptual aftereffects of gaze direction adaptation are unlikely to be explained simply by habituation to low-level image features [27,30,34]. Moreover, these effects are accompanied by reduced haemodynamic responses in the anterior STS to faces with gaze directed to the same side as the adapting stimulus [25], and similarly direction-dependent modulation of scalp potentials at 250 ms and later [35,36], suggesting that these effects may depend on changes in processing in higher-level visual areas.

*Divisive normalization* is another form of gain control relevant to the population coding of stimulus properties. Normalization occurs when the responses of a sensory neuron are not only driven directly by a stimulus that excites it (e.g. by viewing a vertical orientation), but also modulated in relation to the pooled activity across a wider set of functionally related sensory neurons (e.g. neurons tuned to off-vertical orientations). Interestingly, sensory adaptation and normalization mechanisms may interact, such that habituation of a given sensory neuron's responses may affect how it contributes to the normalization of other sensory neurons [37]. In the computational model of gaze perception developed by Palmer & Clifford [31], perceived gaze direction was coded in terms of the relative activity of cell populations tuned to leftwards, rightwards and direct gaze, normalized to the pooled activation across this channel population. It was found that the operation of normalization predicted a distinctive pattern of changes in perceived gaze direction following adaptation to averted gaze. Specifically, while adaptation of channel sensitivity tended to bias perceived gaze direction away from the direction of the adapting stimulus, the additional effect of adaptation on the normalization mechanism caused the magnitude of perceptual changes to differ in a distinctive way across different test stimulus gaze directions. These model predictions fit well with empirical data [30–32], providing psychophysical evidence for the operation of divisive normalization in the gaze system.

Importantly, this indicates that the psychophysical effects of adaptation to gaze direction can be diagnostic of both changes in channel responsiveness, reflected in the *magnitude* of perceptual aftereffects following adaptation to averted gaze, and normalization, reflected in the *profile* of perceptual aftereffects across different test directions. In the present study, we employ this gaze adaptation paradigm to probe the function of these two forms of gain control in schizophrenia. Specifically, we test the psychophysical effects of adaptation to averted gaze in a group of patients with schizophrenia and a group of non-clinical controls, and fit a model of the sensory coding of gaze direction to these data to estimate both the degree of adaptation of underlying sensory responses and the degree of normalization. This builds upon the existing literature on schizophrenia by examining the function of gain control mechanisms in the context of higher-level and social vision. A further purpose of the current study is to contribute to the emerging literature on gaze perception in schizophrenia. Past research has reported differences in how patients with schizophrenia judge gaze direction (e.g. [38,39]) and how their spatial attention is directed by other people's gaze (e.g. [40,41]). However, the underlying sensory coding of other people's direction of gaze is largely unexplored in this condition. This may be an important complement to the more extensive research on higher-level aspects of social cognition, such as theory of mind [19], which in many contexts relies upon the sensory coding of facial properties like gaze direction [42,43].

## 2. Material and methods

### 2.1. Participants

Participants were 22 adults with a diagnosis of schizophrenia (*M*_age_ = 52.0, s.d. = 8.9, 13 males) and 25 healthy controls (*M*_age_ = 41.3, s.d. = 15.2, 12 males). The recruited groups were closely matched in gender, *χ*^2^ (1, *n* = 49) = 0.22, *p* = 0.64, though the group with schizophrenia were somewhat older on average than the control group, *t*_39_ = 3.0, *p* < 0.01. Most participants completed the National Adult Reading Test as a measure of premorbid intelligence, and the full-scale scores of this measure were closely matched between the participants with schizophrenia (*M* = 109, s.d. = 10, *n* = 21) and controls (*M* = 110, s.d. = 7.7, *n* = 18), *t*_37_ = 0.5, *p* = 0.59. One further participant with schizophrenia completed the experiment but was excluded prior to data analysis as they failed to show a monotonic relationship between perceived gaze direction and stimulus gaze direction at baseline.

Patients were recruited from the Volunteer Schizophrenia Research Register of the Australian Schizophrenia Research Bank [44] and the Macquarie Belief Formation Volunteer Register. Diagnosis of Schizophrenia or Schizoaffective Disorder was confirmed using the Diagnostic Interview for Psychosis [45]. This was completed by either a clinical neuropsychologist with over 10 years of experience working with patients or a clinical psychologist, both with postgraduate qualifications in conducting clinical diagnoses for schizophrenia. Clinical demographics were recorded, and symptom severity was assessed using the Scales for Assessment of Positive and Negative Symptoms [46,47]. Clinical ratings were completed by a PhD-level research assistant who was trained by the clinical neuropsychologist and had initial clinical ratings double-scored during training. All patients were on stable doses of antipsychotic medication and each participant had normal or corrected vision. The mean age of diagnosis was 26.1 years (s.d. = 9.6).

Healthy controls were screened using a structured interview to assess for potential affective, psychotic or substance abuse disorders. This was based on the screening modules from the Structural Clinical Interview for Axis 1 Disorders previously outlined under DSM-IV (SCID-1) [48]. Exclusion criteria for both groups included current or past (within the past 5 years) central nervous system disease or head injury, and having less than 8 years of formal education. All participants gave their written informed consent, which was approved by Macquarie University's Ethics Committee (Ref# Caruana5201200021).

### 2.2. Experimental task

**Figure 1.**
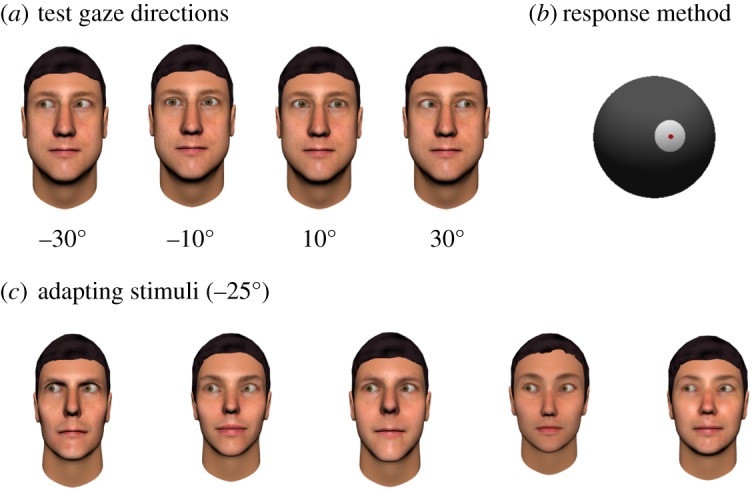
(*a*) Examples of face stimuli with eye gaze direction averted 10° and 30° to the left and right. (*b*) Participants reported the gaze direction of the face stimuli by rotating a spherical pointer that was displayed on screen. (*c*) Participants were adapted to a particular direction of gaze (either 25° left or 25° right) by being shown a series of face images with the same direction of gaze for 1 min.

Participants completed a psychophysical task designed to assess the effects of sensory adaptation on perceived gaze direction. In this task, participants viewed images of computer-generated faces with different directions of eye gaze (figure 1*a*). The procedure for generating face images with precise directions of gaze relative to the viewer, using three-dimensional graphical modelling software, is described elsewhere [31]. Face images were presented on screen at approximately life-size, corresponding to an inter-pupillary distance of roughly 6.3 cm [49]. Participants viewed the images on a 26-inch monitor (1920 × 1080 pixel resolution) at a distance of 80 cm.

The task consisted of three periods: (i) a pre-adaptation test of perceived gaze direction, (ii) an adaptation period, and (iii) a post-adaptation test of perceived gaze direction.

In the *pre-adaptation test of perceived gaze direction*, participants viewed face images with a direction of gaze that was either 30° leftwards, 10° leftwards, 10° rightwards or 30° rightwards. Each image was shown for 500 ms, following which the participant reported the direction in which the face was looking by setting the position of a spherical pointer that was displayed on screen (figure 1*b*). The pointer could be rotated in the horizontal plane, using the mouse. There were 12 trials shown for each test direction, consisting of three different facial identities.

In the *adaptation period*, participants viewed a series of face images that all had gaze averted 25° to the side (figure 1*c*). The adaptation period lasted 1 min and consisted of 15 images shown in a random order, for 4 s each. Each participant was adapted to either 25° leftwards gaze or 25° rightwards gaze, with the direction of adaptation alternating between participants within each group. Previous studies using this task have found that adapting to leftwards and rightwards gaze produces symmetrical effects [30–32]. Thus, in the present study, the data collected from those adapted to 25° rightwards gaze were flipped in sign such that they could be compared directly to those adapted to 25° leftwards gaze. The face images used as adapters were different facial identities from those used as test images during the pre-adaptation and post-adaptation periods.

To encourage participants to attend to the face images during the adaptation period, they performed a simple detection task based on changes in the iris colour of the face stimuli. Specifically, for 20% of the face images shown during the adaptation period, the irises of the face would flash blue for 200 ms at a random point within the 4 s presentation period. Participants were asked to press a key when this occurred, and were considered to have detected the probe if they made a keypress within 1 s of its onset. The mean performance on this task was similarly high in both groups (93% for controls and 89% for participants with schizophrenia).

In the *post-adaptation test of perceived gaze direction*, participants again reported the gaze direction of the same stimuli shown in the pre-adaptation test. The main difference was that each trial began with a single adapting stimulus (25° averted gaze) shown for 4 s. There was a 200 ms inter-stimulus interval between the presentation of the adapting stimulus and the test stimulus in each trial. This use of ‘top-up’ adaptors is intended to maintain adaptation throughout the post-adaptation period of the task. The adaptation period was also repeated halfway through the post-adaptation period for the same reason. Further details of this task are available in [32].

The data collected for each participant before and after adaptation were their mean pointer response (i.e. perceived gaze direction) for each of the stimulus test directions (30° leftwards, 10° leftwards, 10° rightwards and 30° rightwards). For each stimulus test direction, a *perceptual aftereffect* was calculated as the difference in mean pointer response before and after adaptation.

### 2.3. Psychophysical modelling

A psychophysical model of perceived gaze direction was fitted to the participant data as a method for estimating the strength of adaptation and strength of normalization. The mathematical detail of this model is described elsewhere in [31, pp. 1728–1730; 32, pp. 14–16]. Briefly, the perceived gaze direction is taken to be coded across three sensory channels that are broadly tuned, respectively, to leftwards, rightwards and direct gaze. A given face stimulus will produce a different pattern of activation across this population of channels depending on its gaze direction. The perceived gaze direction is modelled in terms of the relative activity across these sensory channels, normalized to the summed activity across channels. The effects of adaptation to gaze direction are modelled as a reduction in the gain of each channel (i.e. a flattening of each channel's sensitivity function) that is proportional to that channel's baseline sensitivity to the adapting stimulus. Adaptation to averted gaze directions produces selective changes in channel gain (i.e. a greater reduction in gain on channels tuned more strongly to the adapting stimulus), such that the relative activation across channels elicited by a given gaze stimulus will tend to differ before and after adaptation.

**Figure 2.**
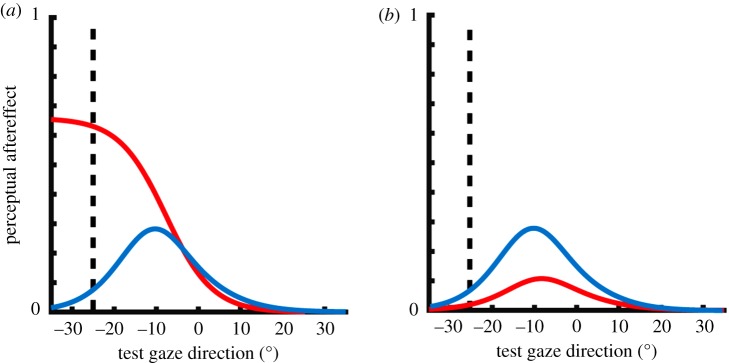
Simulated effects of reduced gain control on the perceptual effects of prolonged exposure to gaze stimuli. The figure shows the predicted size of perceptual aftereffects associated with adaptation to 25° averted gaze, across a range of test stimulus gaze directions. (*a*) The blue line shows predictions by a model where the encoded gaze direction is normalized to the summed activation across sensory channels, while the red line shows perceptual aftereffects predicted by a model with no normalization. As can be seen, reducing the degree of normalization in the model produces a significant change in the profile of perceptual aftereffects such that peak effects occur for test gaze directions more averted than the adapter rather than for test gaze directions less averted than the adapter. (*b*) The red line show predictions by a model with 50% strength of adaptation compared to the blue line. As can be seen, the profile of perceptual aftereffects remains qualitatively similar, but is reduced in magnitude. For more details of this simulation, see [32].

Comparing the direction of gaze computed by the model before and after adaptation results in a set of (predicted) perceptual aftereffects. These modelled aftereffects are fit to the empirical data with the use of three parameters: a *scaling factor*, which maps between the estimated gaze direction computed by the model and the response method used by participants; a *strength of adaptation* parameter (*α*), which captures how strongly sensory adaptation affects channel gain; and a *strength of normalization* parameter (*w*), which captures how strongly individual channel responses are normalized to the activity across the channel population. Figure 2 shows a simulation of the perceptual aftereffects produced by the model for different strengths of normalization (figure 2*a*) and adaptation (figure 2*b*).

By fitting the model to the perceptual aftereffect data observed empirically, we are able to estimate the extent of adaptation and normalization. We first fit the model to the average perceptual aftereffects for each participant group, to estimate the strength of adaptation and normalization exhibited at the group level. We also fit the model to the individual participant data, to estimate the strength of adaptation and normalization exhibited by individual participants. The latter approach allowed us to test statistically for differences between groups in these parameters. The reader is referred to [32, p. 19] for a detailed description of the procedure used in the present study for fitting the model to empirical data.

## 3. Results

### 3.1. Perceptual aftereffects following adaptation to gaze direction

**Figure 3.**
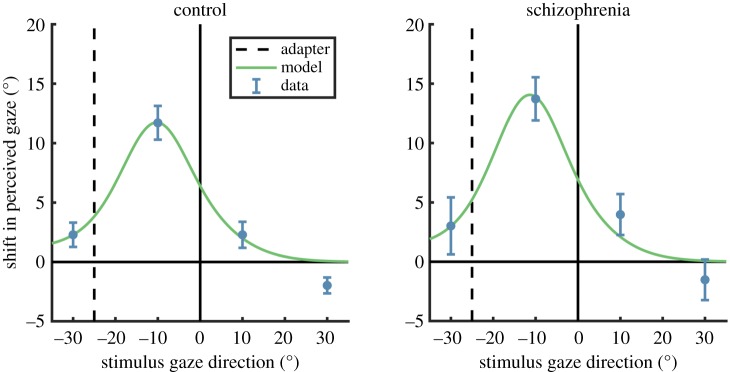
The effect of adaptation to 25° averted gaze on perceived gaze direction, in adults with schizophrenia and healthy controls. The model fits shown here are described in §3.2.

The changes in perceived gaze direction following adaptation to 25° averted gaze are shown in figure 3. As can be seen, both adults with schizophrenia and healthy controls exhibit strong perceptual aftereffects. The magnitude of these perceptual aftereffects and the manner in which their magnitude differs across the stimulus gaze directions tested (i.e. with peak effects occurring between the point of the adapter and the point of direct gaze) closely resemble what we have previously observed in non-clinical samples [30–32].

To test for differences between the participant groups in the effect of sensory adaptation on perceived gaze direction, a three-way mixed analysis of variance (ANOVA) was performed on mean pointer responses (perceived gaze direction). Group (Schizophrenia versus Control) was a between-subjects factor, and Test Direction (−30°, −10°, 10°, 30°) and Block (pre-adaptation versus post-adaptation) were within-subjects factors. There was a main effect of Block, *F*_1,45_ = 97.71, *p* < 0.001, *η*_*p*_^2^ = 0.69, and Test Direction, *F*_3,135_ = 918.13, *p* < 0.001, *η*_*p*_^2^ = 0.95, and a significant interaction between these two factors, *F*_3,135_ = 28.51, *p* < 0.001, *η*_*p*_^2^ = 0.39. However, the two-way interaction between Block and Group, which would indicate a difference in perceptual aftereffects between groups, was not significant, *F*_1,45_ = 2.09, *p* = 0.16, nor was the three-way interaction between Block, Test Direction and Group, *F*_3,135_ = 0.11, *p* = 0.96.

A Bayesian mixed ANOVA was also performed to quantify evidence for the null versus alternative hypotheses. As above, the factors were Group, Block and Test Direction, and the dependent variable was the mean pointer responses. The priors for this analysis were an *r* scale fixed effects of 0.5 and *r* scale random effects of 1. Analyses were performed using the JASP software package [50], and interpreted with respect to guidelines suggested in [51,52]. Note that both BF_10_ and BF_01_ values are reported where appropriate, quantifying evidence for the alternative hypothesis versus the null hypothesis, or the null hypothesis versus the alternative hypothesis, respectively.

The winning model in the ANOVA contained terms for Block, Test Direction, Group, the interaction between Block and Test Direction and the interaction between Group and Test Direction, with decisive support relative to a null model that contained none of the specified factors, BF_10_ = 7.6 × 10^214^. Thus, the winning model did not include an interaction between Group and Block, suggesting no difference in perceptual aftereffects between the groups. In addition, when the interaction terms of interest (i.e. Block × Group, and Block × Test Direction × Group) were tested against a null model that contained all the other specified terms and their interactions, there was moderate evidence for the null model over a model consisting only of the Group×Block interaction term (BF_01_ = 5.5) and very strong evidence for the null model over a model with the additional Group × Block × Test direction interaction term (BF_01_ = 87.1). In sum, the data support there being no difference between participant groups in the effects of sensory adaptation on perceived gaze direction.

### 3.2. Modelling group data

The psychophysical model of perceived gaze direction was first fit to the group-level data. We followed the procedure for model-fitting described previously in [32, p. 19].

The first step was to fit the model of perceived gaze direction to the average baseline data (i.e. pre-adaptation data) for each participant group, to estimate the *scaling factor* parameter that mapped between the perceived direction of gaze computed by the model and the pointer response method used by participants in the task. The best-fitting scaling factors were 44.13 for the control group and 38.78 for the schizophrenia group.

The second step was to incorporate channel adaptation into the model, fitting the predicted effects of adaptation to 25° averted gaze (for the four stimulus gaze directions tested in the experiment) to the average perceptual aftereffects observed in each participant group. The parameters allowed to vary were the strength of adaptation (*α*) and the strength of normalization (*w*). The model fitted the data very well in both participant groups, accounting for 97% of the variance in both cases. These model fits are shown in figure 3.

For the control group, the best-fitting parameters were *α* = 0.66 and *w* = 0.01. For the schizophrenia group, the best-fitting parameters were *α* = 0.79 and *w* = 0.01. Larger *α* values indicate a stronger effect of adaptation on channel sensitivities; thus, there was a trend towards *greater* adaptation in the clinical group compared to the control group (as is also apparent from eye-balling the data in figure 3). The degree of adaptation shown in both groups is comparable to that observed previously in non-clinical groups (*α* = 0.51–0.61) [30–32]. The normalization parameter (*w*) can range from 0 to 1, with a value of 0 indicating that channel responses are fully normalized to the summed activity across channels, and a value of 1 indicating no normalization. Thus, both groups showed evidence for strong normalization of the channel responses.

### 3.3. Modelling individual subjects

The model was also fitted to the data for individual subjects, using the same procedure followed in §3.2, to estimate the scaling factor, strength of adaptation (*α*) and strength of normalization (*w*). This allowed us to test statistically for differences between groups in these parameters. The best-fitting model parameters are summarized for each participant group in table 1.

**Table 1.** Group differences in model parameters.

	controls		schizophrenia				
best-fitting model parameters	mean	s.d.	mean	s.d.	*t* (d.f.)	*p*-value	BF_01_
*α*	0.60	0.27	0.62	0.44	−0.24 (45)	0.81	3.36
*w*	0.11	0.25	0.21	0.38	−1.01 (45)	0.30	2.21
scaling factor	44.13	7.03	38.78	9.23	2.25 (45)	0.03*	0.46
variance explained (%)	75%	25%	66%	31%	0.94 (45)	0.35	2.41

*p < 0.05.

One-sample *t*-tests indicated that the mean strength of adaptation, *α*, was above zero for both the control group, *t*_24_ = 11.1, *p* < 0.001, Hedges *g_rm_* = 3.1, and the schizophrenia group, *t*_21_ = 6.7, *p* < 0.001, Hedges *g_rm_* = 2.0. Similarly, one-sample *t*-tests indicated that the mean strength of normalization, *w*, was below 1 for both the control group, *t*_24_ = −17.9, *p* < 0.001, Hedges *g_rm_* = 5.0, and the schizophrenia group, *t*_21_ = −9.9, *p* < 0.001, Hedges *g_rm_* = 2.9. Thus, both groups showed evidence for significant adaptation and normalization of sensory responses, reflected in how the perceived gaze direction of the test stimuli changed following the adaptation period.

The groups did not differ significantly in the mean strength of adaptation or normalization, consistent with the analyses reported in §3.1. The average model parameters for each group and independent samples *t*-tests are reported in table 1. Bayesian independent samples *t*-tests indicated ‘anecdotal’ or ‘moderate’ support for the null hypothesis that there was no difference in the strength of adaptation or normalization parameters between groups. Bayes factor values are reported in table 1, and are calculated with a Cauchy prior width of 0.7.

## 4. Discussion

In the present study, we aimed to test the function of sensory mechanisms that underlie *perceived gaze direction* in schizophrenia, as a higher-level aspect of visual processing related to social experience. We measured how the perception of gaze direction was affected by recent sensory history, using a perceptual adaptation paradigm in which participants are repeatedly shown faces with a specific direction of gaze. Based on an approach that we have used in healthy controls and adults with autism, we assessed for differences in schizophrenia in the function of two forms of gain control. This was done by employing a computational model of how the perceived direction of gaze is encoded across a set of gaze-selective sensory channels (e.g. cells tuned to faces with distinct directions of gaze). This approach builds upon evidence for cell populations in higher-level visual pathways in the temporal cortex that are selective to the gaze direction of a seen face [23,25,53], and recent computational modelling of psychophysical data that suggests that the perceptual effects of adaptation to gaze direction can be revealing about the functional architecture of this system [30–33]. First, we estimated how the gain on gaze-selective sensory channels is modulated by the recent history of sensory stimulation, reflected in the *magnitude of perceptual aftereffects* induced by adaptation to faces with averted gaze. Second, we estimated how the encoded gaze direction is modulated in relation to the pooled activation across a population of sensory channels (i.e. divisive normalization), reflected in the *profile of perceptual aftereffects* across test stimuli with different gaze directions. The perceptual aftereffects exhibited by patients with schizophrenia provide a clear picture of adaptation and normalization occurring robustly in the context of gaze processing, speaking to the typical coding of gaze direction in the visual system.

### 4.1. Gain control in schizophrenia

These results add to the literature on gain control mechanisms in schizophrenia, which has so far focused mainly on spatial-surround effects in low-level vision (e.g. luminance, contrast, orientation, motion, etc.) [1,13,14]. Our approach differs from this past literature in two main ways. First, we examine a social aspect of perception, tied to the function of higher-level visual pathways. Specifically, the basic coding of the direction of gaze of an observed face appears most closely linked to cortical activity in anterior STS in the temporal lobe, as evidenced by single-cell recording in macaque monkeys and fMRI in humans (see [53], for review), including the effects of adaptation to gaze direction on haemodynamic responses [25]. By contrast, the most consistent findings in the literature on gain control function in schizophrenia relate to contrast-based surround suppression [10], which are linked to a suppression of haemodynamic responses in V1 [11,16]. A partial conclusion of this literature so far is that cortical rather than pre-cortical (i.e. luminance-related) mechanisms are implicated in schizophrenia, particularly related to contrast surround suppression in early visual cortex, with more mixed evidence for differences in visual perception that may reflect function in later visual areas, such as motion-based surround suppression [13,14]. The present results add to this emerging picture by suggesting that cortical gain control in even higher-level visual pathways (e.g. anterior STS) can occur robustly in schizophrenia. Second, gain control is an umbrella term for a range of different modulatory processes, and a distinction can be made between the forms of gain control examined in different studies. In the present study, the mechanisms that we model are the adjustment of the gain on sensory channels (e.g. sensory neurons) based on the temporal context and normalization to the pooled activation across channels with different tuning to gaze direction. By contrast, previous research has focused more commonly on perceptual sensitivity to the spatial context.

Sensory adaptation, whereby neural and perceptual responses to a given stimulus are dependent on the very recent history of sensory stimulation, is a widespread property of the sensory system [7,54]. Perceptual aftereffects are observed for a range of both lower-level sensory properties (e.g. colour, motion, shape) and higher-level sensory properties (e.g. gaze direction, facial expression). The perceptual effects of adaptation have not been comprehensively studied in schizophrenia, such that there is not yet a clear picture in this regard. Calvert *et al*. [55] report differences in how the perceived orientation of a grating is affected by prior exposure to a differently oriented grating (i.e. the ‘tilt aftereffect’); however, only a small sample of individuals were tested and the direction of the differences between those with schizophrenia and controls depended on stimulus parameters and the medication state of the patients. A review of early studies examining the motion aftereffect in schizophrenia concluded that there is evidence for a *longer duration* of the perceptual aftereffect following a period of motion stimulation [56]. One experiment examining a form of colour adaptation (the McCollough effect) found *reduced* adaptability of patients with schizophrenia, in the sense that they needed longer exposure to the adapting stimulus before reporting the expected effect on subsequent perception of colour [57]. More extensively studied is how evoked responses to auditory, visual or tactile stimuli, typically recorded with electroencephalography, change as a stimulus is repeated. In this area of research, findings tend to indicate that neural responses are less strongly modulated with respect to the recent temporal context in schizophrenia relative to controls (e.g. [58–60]). In the present study, both patients with schizophrenia and healthy controls showed strong perceptual adaptation to gaze direction. A Bayesian analysis indicated that there was ‘moderate’ evidence in the data for there being no difference in the magnitude of aftereffects between the groups. Moreover, the schizophrenia group trended towards displaying *stronger* perceptual aftereffects than the control group (figure 3), so there was no suggestion of a reduced susceptibility to sensory adaptation in this context.

The effects of adaptation to eye gaze have been examined in two other clinical groups, namely autism spectrum disorder and prosopagnosia. In prosopagnosia, a group of seven individuals demonstrated typical effects of adaptation to eye gaze, despite the impairments in face recognition that define this condition [61]. In autism, two initial studies found a reduced magnitude of aftereffects following adaptation to averted eye gaze in children [62] and adults [63], consistent with evidence for reduced effects of adaptation to other stimulus types, including face identity [64], numerosity [65] and loudness [66]. However, a more recent study found robust adaptation to gaze direction in adults with autism when using a more direct measure of perceived gaze direction and examining the magnitude of these effects across different test gaze directions [32]. The findings of this latter study in adults with autism mirror the results of the present study in participants with schizophrenia.

### 4.2. Perception of eye gaze in schizophrenia

Research on interpersonal functioning in schizophrenia focuses on impairments of emotion recognition and mental-state reasoning; but little is known of more fundamental perceptual abilities such as those needed to process eye gaze. Perhaps the most prominent finding to date is the evidence for a wider ‘cone of direct gaze’ in schizophrenia. In general, there are a range of eye deviations around direct gaze that we tend to judge as looking directly at us; for instance, in an experiment reported by Mareschal *et al*. [67], non-clinical participants tended to judge gaze deviations of ±3° as looking direct rather than leftwards or rightwards. Several studies have found an increased tendency in schizophrenia to judge averted gaze directions as looking direct [38,39]. Whether this bias is a consequence of beliefs about ‘being watched’ or caused by lower-level perceptual deficits that abet the formation of such delusions is unknown. This finding was not replicated in a recent study that asked participants to report when the face was looking ‘straight ahead’ rather than ‘looking at them’, with this methodological difference perhaps encouraging judgements based more on the geometric direction of gaze rather than self-referential judgements [40]. Thus, one possibility is that the visual perception of gaze direction occurs normally in schizophrenia, but that what differs is judgements about whether a given gaze direction is likely to indicate that one is the focus of another person's attention.

Another key aspect of gaze processing that has been examined in schizophrenia is how one's focus of visual attention is reflexively guided on the basis of another person's direction of gaze. Here, the findings are also somewhat mixed, with some studies finding that patients with schizophrenia are less likely to redirect their attention in response to pictorial eye gaze cues [41,68], but other studies using photographic face images finding similar or even enhanced attentional responses in schizophrenia to other people's direction of gaze or head orientation [40,69–71].

Computational simulations of the effects of adaptation to averted gaze on the encoded gaze direction in the visual system suggest that perceptual aftereffects can be indicative of three distinct characteristics of sensory coding, namely (i) the flexible adjustment of gain on individual sensory channels in relation to the recent history of stimulation, (ii) the divisive normalization of the encoded gaze direction to the pooled activation across sensory channels, and (iii) the channel structure itself (e.g. opponent coding, broadband multichannel coding or narrowband multichannel coding) [31,32]. Thus, the very typical perceptual effects of adaptation to averted gaze that we observe in schizophrenia in the present study, both in the magnitude of perceptual aftereffects and in the tuning of perceptual aftereffects across different test gaze directions, are a strong testament to the typical coding of gaze direction in the visual system in this condition.

In another recent experiment, we examined *perceptual integration* of different facial cues to gaze direction in the same group of individuals with schizophrenia reported in the current paper [72]. In general, our sense of where another person in looking depends on multiple sensory features of their face, including the deviation of their eyes and the orientation of their head [73,74]. Individuals with schizophrenia display this effect as robustly as non-clinical controls, suggesting that the visual integration of facial features that underlies gaze perception is intact in this group. Together with the results of the present paper, these experiments suggest that several basic sensory mechanisms that underlie gaze perception, a key aspect of face processing, are unaffected in schizophrenia.

This research on the sensory mechanisms underlying the perception of gaze direction forms an important complement to research on higher-level social–cognitive impairments in schizophrenia, including the recognition of emotional facial expressions and ‘theory of mind’ [19]. The ability to detect and interpret other people's direction of gaze is a crucial aspect of social cognition, allowing us to share attention with others and understand their intentions, and, in this way, disruption of the sensory coding of gaze direction has the potential to contribute significantly to social–cognitive and social–behavioural difficulties [42,75]. The patients with schizophrenia who completed the present study displayed reduced performance on a theory of mind task compared to controls, in which they were presented with a series of written stories and required to make inferences about the mental states (e.g. false beliefs) of the characters in these stories. These data are reported in the electronic supplementary material. Thus, the evidence for robust gaze processing that we report in the present study for the schizophrenia group occurred despite detectable impairments in social cognition.

### 4.3. Conclusion

The perceptual processing of other people's gaze direction is largely unexplored in schizophrenia. Data in this regard are useful for two reasons. First, gaze processing is a key aspect of social cognition and social behaviour, and understanding gaze perception in schizophrenia helps to build a profile of the social cognitive strengths and impairments that exist in this condition. Our findings of strong perceptual aftereffects and our modelling analysis of these data suggest that key functional mechanisms important to the basic sensory coding of other people's direction of gaze in the visual system are operating robustly in schizophrenia. This includes the flexible adjustment of channel gain in response to the recent sensory history and the divisive normalization of sensory responses. Second, past research has examined the operation of gain control mechanisms across different modalities of lower-level vision (e.g. luminance, contrast, motion, size), with apparently *impaired gain control* being evident for some but not all of these processes [1,13,14]. Gaze processing is a key example of higher-level and social visual perception that rests in part upon comparable perceptual mechanisms to lower-level vision, such as the population coding of stimulus parameters and flexible adjustment of sensory gain. The evidence that we report for intact gain control in the context of gaze perception thus adds to the emerging picture of how sensory processing differences are distributed across the visual system in schizophrenia.

## Supplementary Material

Supplementary methods and results

## Data accessibility

Data are accessible at https://osf.io/nvjct/?view_only=b1c466142e2d4bb0abd3dcd9fe6506f7.
