# Supplementary material for: Adaptive sensory coding of gaze direction in schizophrenia
Source: R Soc Open Sci. 2018 Dec 12;5(12):180886. doi: 10.1098/rsos.180886 (PMC6304156; doi:10.1098/rsos.180886)
Supplement: Supplementary methods and results [file rsos180886supp1.docx]

**Supplementary Material for the article “Adaptive sensory coding of gaze direction in schizophrenia” in Royal Society Open Science**

Colin J. Palmer*^1^, Nathan Caruana*^2, 3^, Colin W.G. Clifford^1^, Kiley Seymour^2,3, 4^

^1^ School of Psychology, UNSW, Sydney, NSW 2052, Australia

^2^ Department of Cognitive Science, Macquarie University, Sydney, NSW 2109, Australia

^3^ ARC Centre of Excellence for Cognition and its Disorders, Australia

^4^ School of Social Sciences and Psychology, Western Sydney University, Sydney, NSW 2150, Australia

* These authors contributed equally

Correspondence can be addressed to Colin Palmer at [Colin.Palmer@unsw.edu.au](mailto:Colin.Palmer@unsw.edu.au) or School of Psychology, UNSW Sydney, NSW 2052, Australia.

**Theory of mind task**

Participants also completed a task to assess higher-order social cognitive function. Impaired performance on tasks that require inferring or reasoning about other people’s mental states has been reported previously in samples with schizophrenia [1]. In the present study, participants read a series of written stories, and were asked questions about the characters in these stories that were designed to assess ‘first order’ theory of mind (e.g., understanding that a character can have a false belief), ‘second order’ theory of mind (e.g., understanding one character’s beliefs about another character’s beliefs), and general comprehension or attention to the story. The questions related to both how a character was likely to act next in the story (scored as 1 or 0 for correct or incorrect responses) and why they would act in this way (scored as 2, 1, or 0 for correct, partially correct, or incorrect responses). There were four stories in total, including two focussed on first-order theory of mind and two focussed on second-order theory of mind. The comprehension questions were scored as 1 or 0 for correct or incorrect responses. These scores were summed across both stories within each story condition (i.e., first-order, second order), resulting in a maximum score of six for theory of mind questions and two for comprehension questions in each story condition. For further details, see [2] and [3].

**Theory of mind task results**

There was a significant group*condition interaction, *F*(1,45) *=* 8.30, *p* = .006, in which the schizophrenia group performed significantly poorer that controls for both first order theory of mind questions, *t*(28) = -2.84, *p* = .0018, and second order theory of mind questions, *t*(45) = -4.24, *p* < .001, but not on comprehension questions (*p*s > .16). Descriptive statistics for this task are presented in **Supplementary Table 1**.

**Supplementary Table 1**. Means and standard deviations for theory of mind task

| Stories | Schizophrenia group | Control group |
| --- | --- | --- |
| Theory of mind, first order | 4.18 (2.11) | 5.56 (0.92) |
| Theory of mind, second order | 2.86 (1.83) | 4.88 (1.42) |
| Comprehension, first order | 1.73 (0.55) | 1.88 (0.33) |
| Comprehension, second order | 1.91 (0.29) | 2.00 (0.00) |

The sample of participants that completed the present study largely overlaps with those that completed another experiment that will be reported elsewhere [4], and, correspondingly, the theory of mind task results reported here and in that manuscript are largely the same data, rather than separate replications.

**References**

1 Green, M. F., Horan, W. P., Lee, J. 2015 Social cognition in schizophrenia. *Nature reviews. Neuroscience*. **16**, 620-631. (10.1038/nrn4005)

2 Langdon, R., Connors, M. H., Connaughton, E. 2014 Social cognition and social judgment in schizophrenia. *Schizophr Res Cogn*. **1**, 171-174. (10.1016/j.scog.2014.10.001)

3 Harrington, L., Langdon, R., Siegert, R. J., McClure, J. 2005 Schizophrenia, theory of mind, and persecutory delusions. *Cogn Neuropsychiatry*. **10**, 87-104. (10.1080/13546800344000327)

4 Palmer, C. J., Caruana, N., Clifford, C. W. G., Seymour, K. Under Review Perceptual integration of head and eye cues to gaze direction in schizophrenia.
